# Mortality Risk of Antidiabetic Agents for Type 2 Diabetes With COVID-19: A Systematic Review and Meta-Analysis

**DOI:** 10.3389/fendo.2021.708494

**Published:** 2021-09-16

**Authors:** Chengxia Kan, Yang Zhang, Fang Han, Qian Xu, Tongtong Ye, Ningning Hou, Xiaodong Sun

**Affiliations:** ^1^Department of Endocrinology, Affiliated Hospital of Weifang Medical University, Weifang, China; ^2^Clinical Research Center, Affiliated Hospital of Weifang Medical University, Weifang, China; ^3^Department of Pathology, Affiliated Hospital of Weifang Medical University, Weifang, China

**Keywords:** COVID-19, diabetes, antidiabetic agents, mortality, type 2 diabetes

## Abstract

**Aims:**

We conducted a systematic review and meta-analysis to assess various antidiabetic agents’ association with mortality in patients with type 2 diabetes (T2DM) who have coronavirus disease 2019 (COVID-19).

**Methods:**

We performed comprehensive literature retrieval from the date of inception until February 2, 2021, in medical databases (PubMed, Web of Science, Embase, and Cochrane Library), regarding mortality outcomes in patients with T2DM who have COVID-19. Pooled OR and 95% CI data were used to assess relationships between antidiabetic agents and mortality.

**Results:**

Eighteen studies with 17,338 patients were included in the meta-analysis. Metformin (pooled OR, 0.69; *P*=0.001) and sulfonylurea (pooled OR, 0.80; *P*=0.016) were associated with lower mortality risk in patients with T2DM who had COVID-19. However, patients with T2DM who had COVID-19 and received insulin exhibited greater mortality (pooled OR, 2.20; *P*=0.002). Mortality did not significantly differ (pooled OR, 0.72; *P*=0.057) between DPP-4 inhibitor users and non-users.

**Conclusions:**

Metformin and sulfonylurea could be associated with reduced mortality risk in patients with T2DM who have COVID-19. Furthermore, insulin use could be associated with greater mortality, while DPP-4 inhibitor use could not be. The effects of antidiabetic agents in patients with T2DM who have COVID-19 require further exploration.

**Systematic Review Registration:**

PROSPERO (identifier, CRD42021242898).

## Introduction

Coronavirus disease 2019 (COVID-19), caused by SARS-CoV-2, is a serious global public health problem that has affected more than 100 million people worldwide (as of January 26, 2021) (1). SARS-CoV-2 exhibits high infectivity, causing rapid spread and associated outbreaks; moreover, the mortality of COVID-19 was 61.5% at the onset of the epidemic (2). Although mortality rates have declined with increasing disease awareness, poor outcomes persist, especially among people with chronic diseases (3). Diabetes, characterized by hyperglycemia, is an increasingly common illness worldwide; it is associated with the onset of various complications, including infections. Richardson et al. found that 42% of patients with COVID-19 had diabetes (defined as HbA1c level >6.5%) (4). Patients with diabetes, especially those who have COVID-19, are vulnerable to the onset of severe disease. In a study of 1000 COVID-19 patients, diabetes was present in 16.2% of patients with severe disease; the final outcomes of these patients were mechanical ventilation and/or mortality (5).

Among patients with diabetes or hyperglycemia, the in-hospital mortality is reportedly 29%, compared with 6% among people without diabetes or hyperglycemia (4). This indicates a fourfold increase in COVID-19 mortality among patients with diabetes or hyperglycemia. In a retrospective study of 72,314 COVID-19 patients, the mortality rate (7.3%) was significantly greater in patients with diabetes (6). Crouse et al. (7) found that increased mortality persisted in patients with diabetes who had COVID-19, despite adjustment for covariates such as age, ethnicity, obesity, and hypertension. Sourij H et al. (8) also found that in-hospital mortality for COVID-19 was higher in patients with diabetes.

Glycemic control is critical in diabetes mellitus patients with COVID-19 because hyperglycemia can increase mortality in these patients. Type 2 diabetes mellitus (T2DM), which is present in most individuals with diabetes, is characterized by insulin hyposecretion and insulin resistance. The primary treatment involves administration of oral antihyperglycemic drugs or insulin. Several reports have investigated mortality among patients with T2DM who have COVID-19 that receive treatment with antihyperglycemic therapy. Previous meta-analysis has done the use of metformin in diabetes since the early pandemic time (9). However, this study is still limited in the included study. In addition, due to variations in experimental design, final research outcomes, and patient populations, results are inconsistent among studies. Accordingly, we conducted a systemic review and meta-analysis to investigate the associations of antidiabetic agents with mortality in patients with T2DM who have COVID-19.

## Methods

### Search Strategy

We performed a comprehensive literature search from the date of inception until February 2, 2021 in four databases: PubMed, Web of Science, Embase, and the Cochrane Library. The search strategy used the following specific keywords: “COVID-19” OR “coronavirus” OR “SARS-CoV-2 infection” OR “2019 novel coronavirus disease” AND “diabetes mellitus, type 2” OR “diabetes, type 2” OR “type 2 diabetes” AND “antidiabetic agents” OR “metformin” OR “sulfonylurea compounds” OR “glucagon-like peptide 1” OR “dipeptidyl-peptidase IV inhibitors” OR “sodium-glucose transporter 2 inhibitors” OR “glycoside hydrolase inhibitors” OR “Insulin”. After screening to identify relevant abstracts, we searched the reference lists of retrieved articles to discover additional potentially eligible studies. This study was performed in accordance with the Preferred Reporting Items for Systematic Reviews and Meta-analyses (PRISMA) guidelines (10). Two reviewers independently evaluated all articles that met the requirements (described in Section 2.2) and extracted data, then compared their findings. Any differences were resolved through discussion.

### Inclusion and Exclusion Criteria

The search strategy was based on a Population, Intervention, Comparison, and Outcome (PICO) framework. Population: patients with T2DM who had COVID-19; Intervention: any anti-diabetic agent; Comparison: without anti-diabetic agent usage; Outcome: study type, mortality.

Inclusion criteria were as follows: study type, published studies regarding associations between antidiabetic agents and patients with T2DM who had COVID-19; exposure intervention, patients with T2DM who had COVID-19 received antidiabetic agents (e.g., metformin, sulfonylurea, glucagon-like peptide 1 [GLP-1] receptor agonists, dipeptidyl peptidase 4 [DPP-4] inhibitors, sodium-glucose cotransporter 2 [SGLT2] inhibitors, α-glucosidase inhibitors, and/or insulin); and outcome indicator, quantitative assessment of associations between antidiabetic agent use and patient mortality, including odds ratios (ORs) and 95% confidence intervals (CIs).

Exclusion criteria were as follows: study type, non-research/original articles (e.g., review articles, case reports, case series, summaries of meetings or discussions, or letters to the editor); incomplete patient information; duplicate/overlapping data; and absence of mortality outcomes.

### Data Extraction and Quality Assessment

Following application of the inclusion and exclusion criteria, two investigators (Kan CX and Hou NN) independently collected data from the 18 identified studies, using a standardized method. The following data were extracted from enrolled studies: first author surname, type of study, country of origin, and sample size; clinical data including age and nationality; and statistical data (e.g., OR or HR and corresponding 95% CI values). When both univariate and multivariate analyses were reported, multivariate data were included in the analysis. If the OR was not reported directly, relevant data were used to calculate the OR.

Article quality was evaluated using the Newcastle-Ottawa Scale tool (11). A Newcastle-Ottawa Scale score ≥7 was considered high-quality; higher Newcastle-Ottawa Scale scores were presumed to indicate higher literature quality.

### Statistical Analysis

Meta-analysis was carried out using Stata software, version 16.0. Pooled OR and 95% CI values were used to estimate relationships between antidiabetic agents and mortality. Heterogeneity was evaluated using the Cochran Q statistic and the I^2^ statistic. A random-effects model (*P*<0.1 or I^2^≥50%) and a fixed-effects model (*P*>0.1 and I^2^<50%) were used to assess heterogeneity. If >5 studies were included, Egger’s test and Begg’s test were used to evaluate publication bias (12). *P*>0.05 was considered to indicate the absence of publication bias.

## Results

### Study Selection

**Figure 1:**
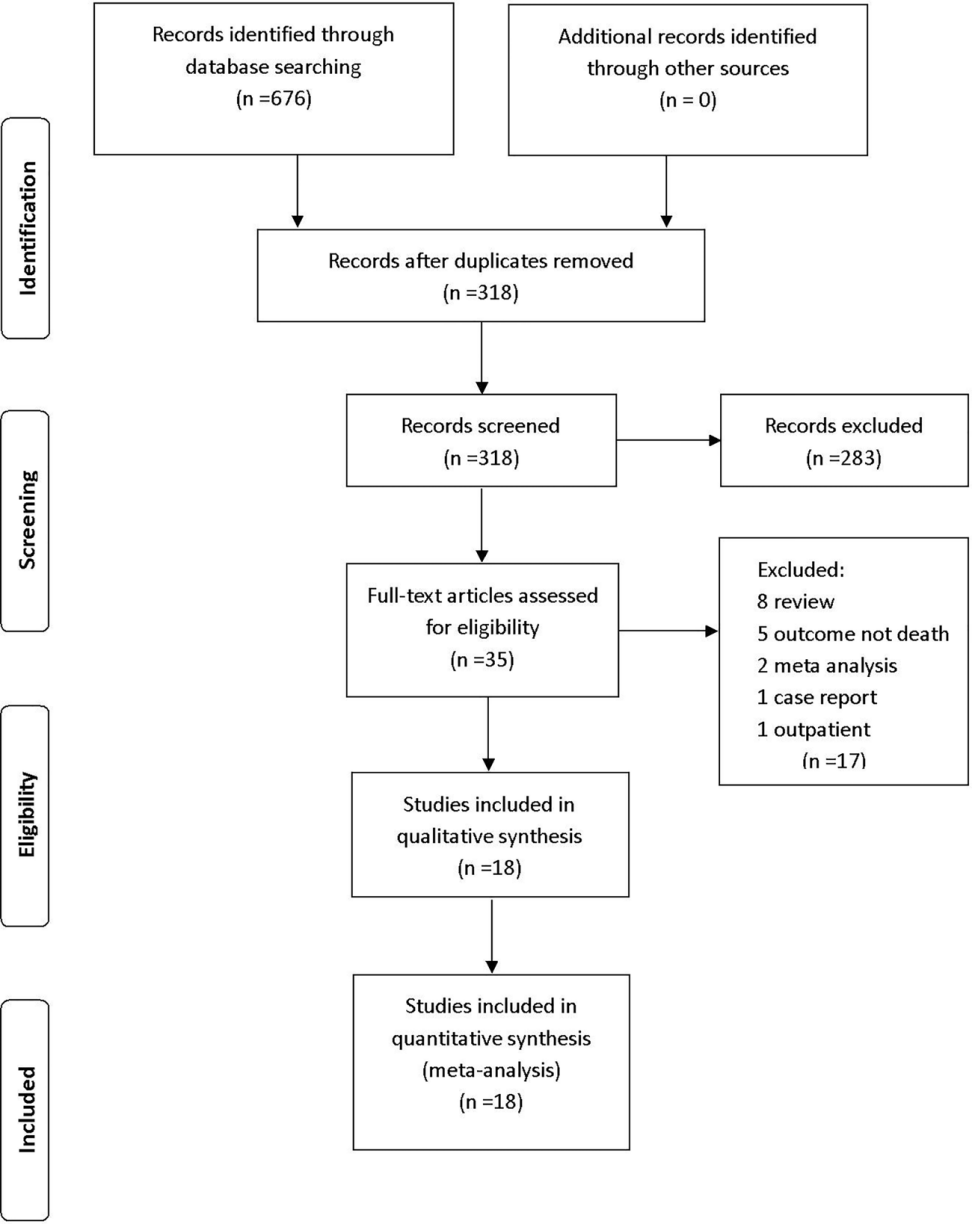
PRISMA study.

We retrieved 676 studies from PubMed, Web of Science, Embase, and the Cochrane Library database. After the removal of duplicates, 318 articles were included in title and abstract screening; 283 articles were excluded on the basis of abstract or title. The remaining 35 articles were included in the full-text review; 17 studies were then excluded (eight reviews, five studies that did not report mortality, two meta-analyses, one case report, and one study that included outpatients). The remaining 18 eligible studies were included in the analysis (**Figure 1**).

### Study Characteristics and Quality Assessment

We investigated 18 studies that included 17,338 patients with T2DM who had COVID-19. Among these 18 studies, seven were performed in China, two were performed in the United States, one was performed in South Korea, two were performed in the United Kingdom, three were performed in Italy, and three were performed in France. All Newcastle-Ottawa Scale scores were ≥7 (**Table 1**).

**Table 1:** The characteristics of the included studies in meta-analysis.

Study	Study type	Country	All Subjects	Patients (n)	Ages (years)	Mortality	Odds ratio/Hazard ratio	NOS
					Mean±SD/median (IQR)		(95% CI)	
Metformin users/non users								
Chen et al. (13)	Retrospective	China	120	43/77	62.0 (56.0-69.0) *vs* 67.0 (57.5-73.0)	4 (9.3%) *vs* 15 (19.5%)	0.62 (0.17-2.20)	8
Bramante et al. (14)	Retrospective	USA	6256	2333/3923	73 *vs* 76	394 (16.9%) *vs* 791 (20.2%)	Female subgroup 0.74 (0.56- 0.98)	8
Luo et al. (15)	Retrospective	China	283	104/179	63.0 (55.8-68.3) *vs* 65.0 (57.5-71.0)	3 (2.9%) *vs* 22 (12.3%)	4.36 (1.22-15.59)	9
Crouse et al. (7)	Retrospective	USA	220	76/144	N/A	8 (19.1%) *vs* 34 (81.0%)	0.33 (0.13-0.84)	8
Kim et al. (16)	Retrospective	South Korea	235	113/122	68.3±11.9 (all group)	N/A	0.36 (0.10-1.23)	9
Philipose et al. (17)	Retrospective	UK	159	100/59	N/A	N/A	1.39 (0.84-2.16)	8
Cheng et al. (18)	Retrospective	China	1213	678/535	62.0 (55.0-68.0) *vs* 64.0 (58.0-70.0)	N/A	HR:1.65 (0.71-3.86)	9
Abu-Jamous et al. (19)	Retrospective	UK	191	23/168	N/A	4 (17.4%) *vs* 94 (56.0%)	0.19 (0.05-0.70)	9
Jiang et al. (20)	Retrospective	China	328	100/228	64.0 (56.5, 70.0) *vs* 67.0 (60.0, 76.0)	3 (3.0%) *vs* 25 (11.0%)	0.48 (0.13-1.74)	8
Mirani et al. (21)	Retrospective	Italy	90	69/21	69±13 *vs* 75±8	25 (36.2%) *vs* 13 (61.9%)	0.43 (0.21-0.85)	7
Li et al. (22)	Retrospective	China	131	37/94	64.6 ± 11.2 *vs* 67.7 ± 11.7	2 (5.4%) *vs* 21 (22.3%)	0.20 (0.04-0.90)	8
Lalau et al. (23)	Prospective	France	2449	1496/953	68.5 ± 11.9 *vs* 74.6 ± 12.5	Day 7:122 (8.2%) *vs* 153 (16.1%)	Day 28 0.71 (0.54-0.94)	9
						Day 28:239 (16.0% )*vs* 273 (28.6%)		
Cariou et al. (24)	Prospective	France	1317	746/571	69.8±13.0 (all group)	N/A	0.59 (0.42-0.84)	8
Wargny et al. (25)	Prospective	France	2794	1553/1241	68.9 ± 13.2 (all group)	N/A	0.63 (0.52-0.77)	8
Sulfonylurea users/non users								
Chen et al. (13)	Retrospective	China	120	53/67	66.0 (60.0-72.5) *vs* 64.0 (55.0-73.0)	7 (13.20%) *vs* 12 (17.91%)	0.68 (0.21-2.16)	8
Kim et al. (16)	Retrospective	South Korea	235	60/175	68.3±11.9 (all group)	N/A	0.84 (0.23-3.09)	9
Mirani et al. (21)	Retrospective	Italy	90	Oct-80	75±8 *vs* 70±12	3 (30%) *vs* 35 (43.8%)	0.34 (0.08-1.42)	7
Cariou et al. (24)	Prospective	France	1317	367/950	69.8±13.0 (all group)	N/A	0.74 (0.49-1.13)	8
Wargny et al. (25)	Prospective	France	2794	782/2012	68.9 ± 13.2 (all group)	N/A	0.83 (0.67-1.03)	8
DPP-4 inhibitors users/non users								
Chen et al. (13)	Retrospective	China	120	20/100	66.0 (56.0-73.0) *vs* 65.0 (57.0-72.0)	5 (25.00%) *vs* 14 (14.00%)	1.48 (0.40-5.53)	8
Kim et al. (16)	Retrospective	South Korea	235	85/150	68.3±11.9 (all group)	N/A	1.47 (0.45-4.78)	9
Mirani et al. (21)	Retrospective	Italy	90	Nov-79	70±13 *vs* 71±12	1 (9.1%) *vs* 37 (46.8%)	0.13 (0.02–0.92)	7
Solerte et al. (26)	Retrospective	Italy	338	169/169	69.0 ± 0.9 *vs* 69.0 ± 1.0	31 (18%) *vs* 63 (37%)	0.44 (0.29-0.66)	8
Fadini et al. (27)	Retrospective	Italy	81	Sep-72	72.2 (12.8) *vs* 70.1 (13.3)	1 (11.1%) *vs* 10 (13.9%)	0.77 (0.09-6.88)	8
Zhou et al. (28)	Retrospective	China	444	111/333	63 (55-67) *vs* 64 (56.5-69)	2 (1.8%) *vs* 11 (3.3%)	0.58 (0.12-2.68)	8
Cariou et al. (24)	Prospective	France	1317	285/1032	69.8±13.0 (all group)	N/A	0.85 (0.55-1.32)	8
Wargny et al. (25)	Prospective	France	2794	615/2179	68.9 ± 13.2 (all group)	N/A	0.83 (0.65-1.05)	8
GLP-1 analogs users/non users								
Cariou et al. (24)	Prospective	France	1317	123/1194	69.8±13.0 (all group)	N/A	0.64 (0.32-1.29)	8
Wargny et al. (25)	Prospective	France	2794	254/2540	68.9 ± 13.2 (all group)	N/A	0.78 (0.53-1.15)	8
SGLT-2 inhibitors users/non users								
Kim et al. (16)	Retrospective	South Korea	235	8/227	68.3±11.9 (all group)	N/A	5.05 (0.48-53.26)	9
α-Glucosidase inhibitors users/non users								
Chen et al. (13)	Retrospective	China	120	69/51	66.0 (56.5-73.0) *vs* 65.0 (56.0-72.0)	11 (15.94%) *vs* 8 (15.69%)	0.99 (0.31-3.14)	8
Insulin users/non users								
Chen et al. (13)	Retrospective	China	120	71/49	65.0 (57.0-72.0) *vs* 65.0 (56.0-73.0)	16 (22.5%) *vs* 3 (6.12%)	2.99 (0.67-13.30)	8
Crouse et al. (7)	Retrospective	USA	220	87/133	N/A	15 (35.7%) *vs* 27 (64.3%)	0.97 (0.42-2.23)	8
Kim et al. (16)	Retrospective	South Korea	235	19/216	68.3±11.9 (all group)	N/A	0.26 (0.03-2.63)	9
Mirani et al. (21)	Retrospective	Italy	90	29/61	72 ±10 *vs* 70 ±13	19 (65.5%) *vs* 19 (31.2%)	3.34 (1.74-6.41)	7
Yu et al. (29)	Retrospective	China	689	346/343	67 (58-75) *vs* 65 (56-71)	94 (27.2%) *vs* 12 (3.5%)	7.70 (4.22-14.05)	8
Cariou et al. (24)	Prospective	France	1317	504/813	69.8±13.0 (all group)	N/A	1.71 (1.20-2.43)	8
Wargny et al. (25)	Prospective	France	2796	1039/1757	68.9 ± 13.2 (all group)	N/A	1.72 (1.41-2.08)	8

NA means Not Applicable.

### Relationships of Antidiabetic Agents With Mortality Risk

**Figure 2:**
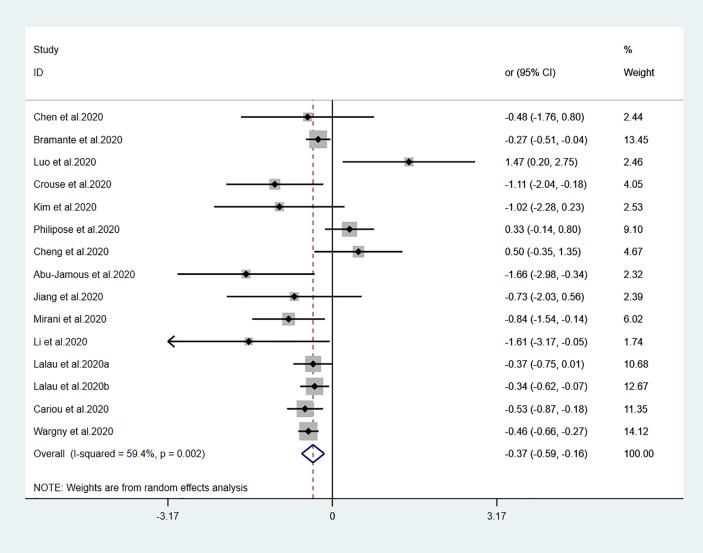
Forest plots of meta-analysis of the relationship between metformin therapy and the risk of mortality in patients with type 2 diabetes who have COVID-19. The diamonds and horizontal lines indicate the corresponding odds ratio and 95% confidence interval. The size of the gray area represents the specific statistical weight of the study. The vertical solid line represents the OR of 1, and the vertical red dotted line shows the combination effect estimation. The suffix “a” or “b” after the studies indicates results of the same study at different times.

**Figure 3:**
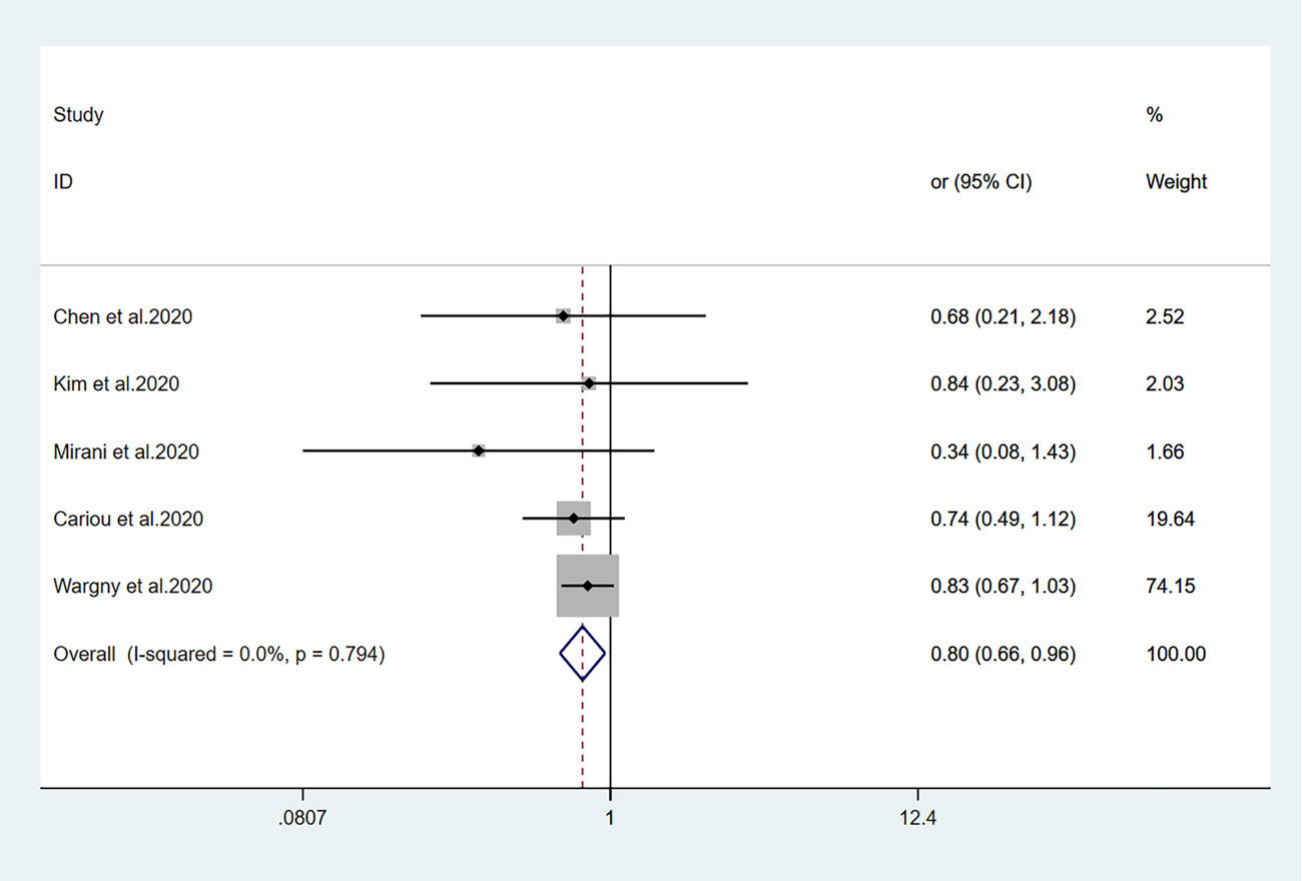
Forest plots of meta-analysis of the relationship between sulfonylurea therapy and the risk of mortality in patients with type 2 diabetes who have COVID-19.

**Figure 4:**
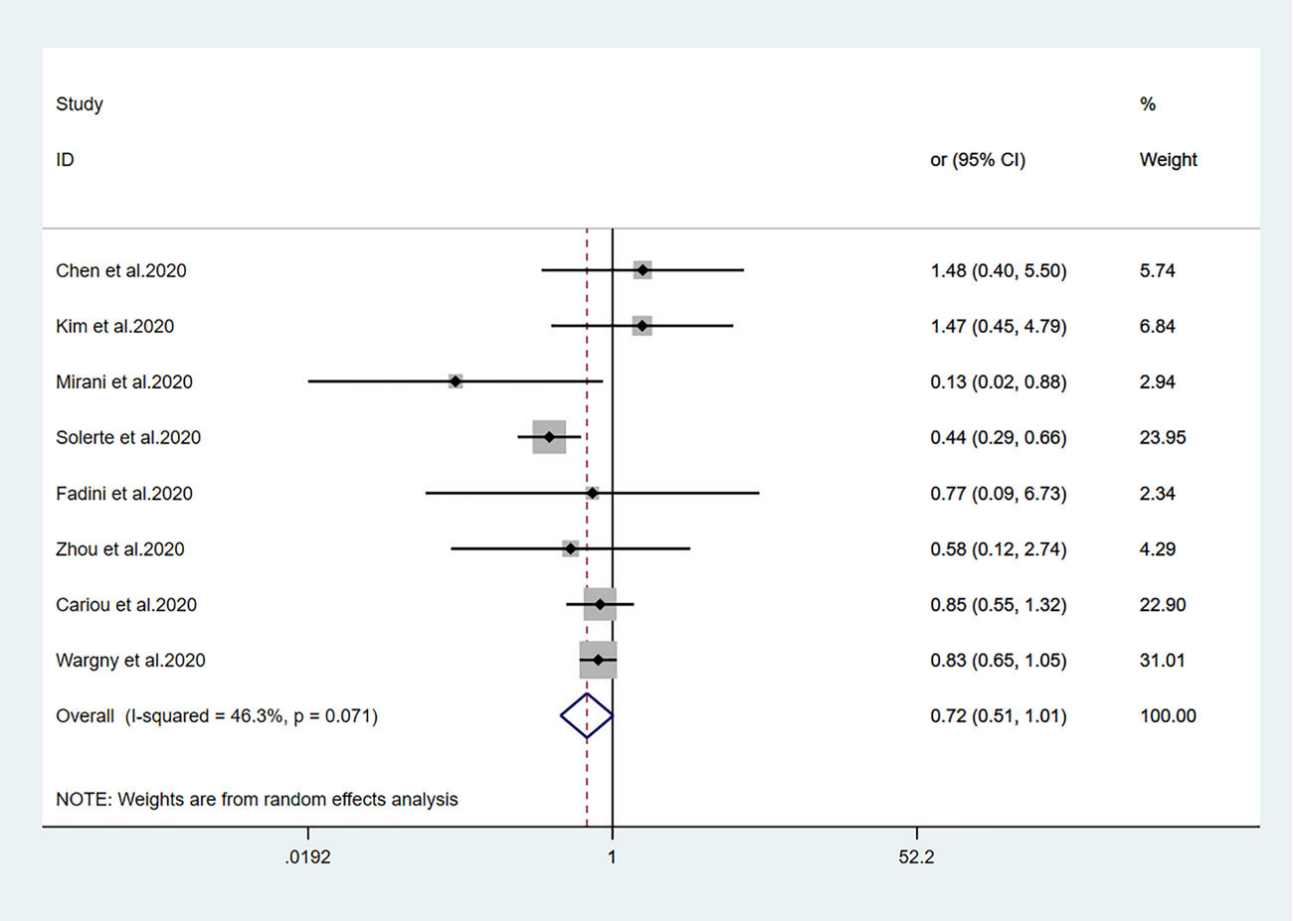
Forest plots of meta-analysis of the relationship between DPP4 inhibitors therapy and the risk of mortality in patients with type 2 diabetes who have COVID-19.

**Figure 5:**
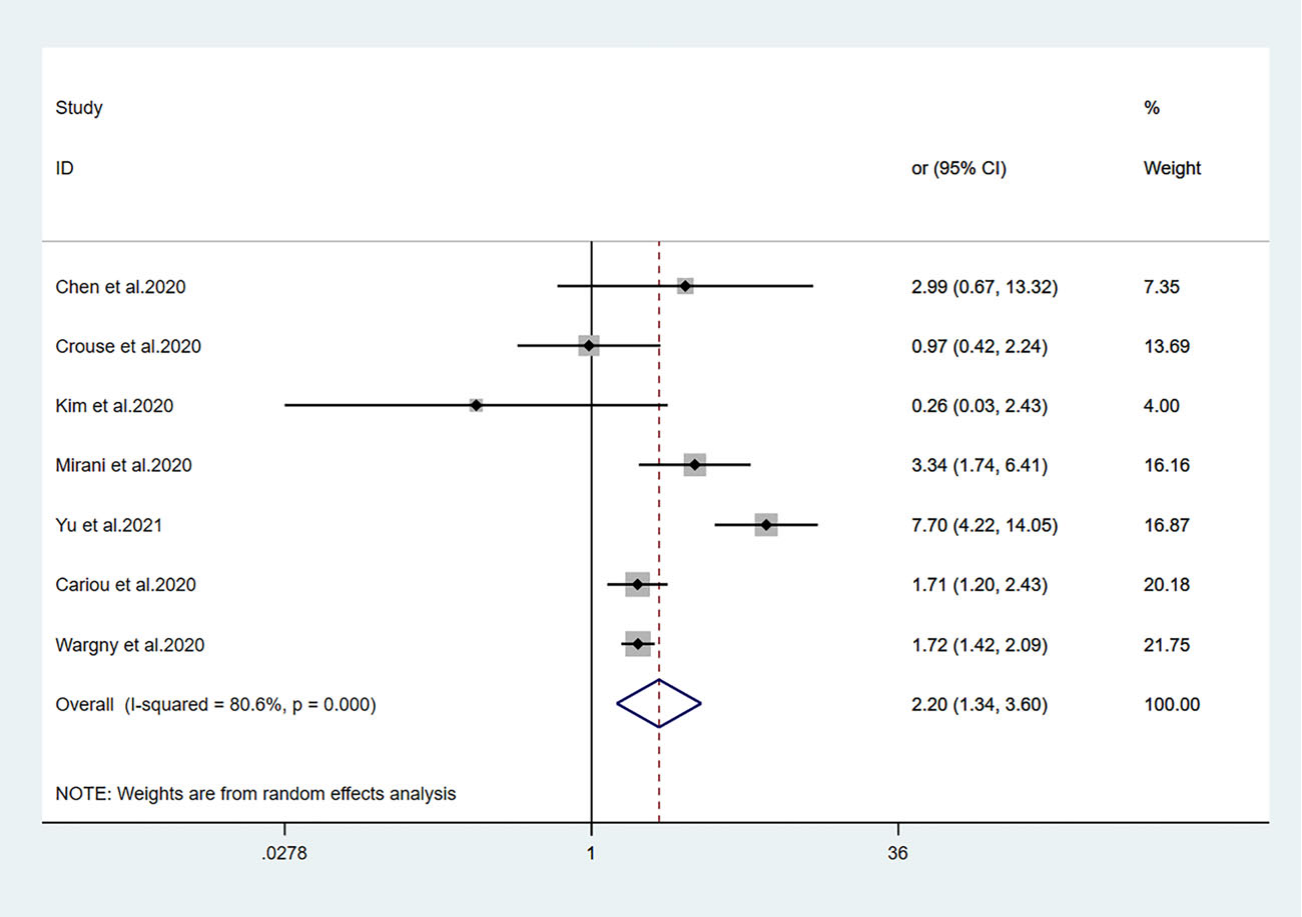
Forest plots of meta-analysis of the relationship between insulin therapy and the risk of mortality in patients with type 2 diabetes who have COVID-19.

The relationships between hypoglycemic drugs and mortality are shown in **Figures 2**–**5**. Because only two studies examined GLP-1 receptor agonists (24, 25), one study examined α-glucosidase inhibitors (13), and one study examined SGLT2 inhibitors (16), the OR values of these studies could not be combined. Significant associations were identified between antidiabetic agent use (i.e., metformin, sulfonylurea, and insulin) and mortality (all *P*<0.05). There was no significant association between DPP-4 inhibitor use and mortality (*P>*0.05).

### Effects of Metformin, Sulfonylurea, DPP-4 Inhibitors, and Insulin on Mortality

Fourteen studies (7, 13–25) focused on metformin users in the meta-analysis. Pooled OR analysis revealed signifi0063antly lower mortality in metformin users than in metformin non-users (**Figure 2**; pooled OR, 0.69; 95% CI, 0.55–0.86; *P*=0.001). Five studies (13, 16, 21, 24, 25) showed lower mortality in sulfonylurea users than in sulfonylurea non-users (**Figure 3**; pooled OR, 0.80; 95% CI, 0.66–0.96; *P*=0.016). Eight studies (13, 16, 21, 24–28) revealed no significant difference in mortality between DPP-4 inhibitors users and non-users (**Figure 4**; pooled OR, 0.72; 95% CI, 0.51–1.01; *P*=0.057). Finally, seven studies (7, 13, 16, 21, 24, 25, 29)showed greater mortality in insulin users than in insulin non-users (**Figure 5**; pooled OR, 2.20; 95% CI, 1.34–3.60; *P*=0.002).

### Publication Bias

Begg’s test and Egger’s test showed no significant publication bias among the included studies (**Table 2**).

**Table 2:** The result of publication bias.

Death events	No. of studies	OR (95% CI)	P-value	Heterogeneity		Model used	Begger’s test	Egger’s test
				I2	P-value			
Metformin	14	0.69 (0.55-0.86)	0.001	59.4%	0.002	Random	0.198	0.745
Sulfonylurea	5	0.80 (0.66-0.96)	0.016	0	0.794	Fixed	0.462	0.180
DPP-4 inhibitors	8	0.72 (0.51-1.01)	0.057	46.3%	0.071	Random	0.386	0.777
Insulin	7	2.20 (1.34-3.60)	0.002	80.6%	0.000	Random	1.000	0.706

## Discussion

This comprehensive meta-analysis included 18 studies with 17,338 subjects. Overall, metformin and sulfonylurea users exhibited lower mortality risk, compared with the respective non-users, among patients with T2DM who had COVID-19. However, patients who received insulin treatment exhibited greater mortality risk, while DPP-4 inhibitor use was not associated with mortality. This is the first study to evaluate the mortality risk associated with the use of various antidiabetic agents among T2DM patients with COVID-19.

Diabetes is associated with a poor outcome that involves Acute Respiratory Distress Syndrome (ARDS), serious clinical manifestations, and mortality (30). Meta-regression analysis showed that, as a single risk factor, diabetes was a stronger influence among younger and non-hypertensive patients; however, this finding requires further investigation. Patients with diabetes are more susceptible to infection, as indicated by Pearson-Stuttard et al. (31). Given the high incidence rate of diabetes worldwide, these people comprise a considerable proportion of individuals with COVID-19. Research regarding influenza A virus (H1N1) (32) in 2009, SARS-CoV (33) in 2002, and Middle East Respiratory Syndrome Coronavirus (MERS-CoV) (34) in 2012 showed that hyperglycemia was an essential predictor for severity and mortality. Researches regarding SARS-CoV-2 pandemic have found that patients with T2DM are at greater risk of mortality from COVID-19 (5, 6, 35). Patients with a more severe course of diabetes have a poorer prognosis of COVID-19 (36). Thus far, there have been multiple studies regarding antihyperglycemic drugs and mortality in T2DM patients with COVID-19. However, the mortality risks associated with the use of each antihyperglycemic drug in these patients remain unclear. The present meta-analysis aimed to assess mortality in those patients.

Compared with metformin non-users, metformin users exhibited lower COVID-19-related mortality in our meta-analysis. Several previous meta-analyses have shown that metformin was associated with lower mortality; these include the studies by Kow et al. (37) (OR, 0.62; 95% CI, 0.43–0.89), Scheen et al. (38) (OR, 0.75; 95% CI, 0.67–0.85), and Lukito et al. (39) (OR, 0.64; 95% CI, 0.43–0.97) and Hariyanto et al. (9) (RR, 0.54; 95% CI, 0.32–0.90). Our meta-analysis revealed that metformin use was associated with lower mortality risk in T2DM patients with COVID-19, similar to previous findings. We presume that metformin reduces the mortality risk through two potential mechanisms. First, metformin can mediate an anti-inflammatory effect (40, 41). In particular, metformin can reverse pulmonary fibrosis, delay ARDS progression, improve patient prognosis, and reduce mortality (42). There is increasing evidence that the Interleukin-6 (IL-6) signaling pathway can be inhibited by metformin; specifically, metformin substantially reduces the expression of IL-6 receptors and promotes myeloma cell apoptosis in patients with primary myeloma (43). Polycystic ovary syndrome patients reportedly exhibit lower serum IL-6 levels and improved chronic inflammation during early use of metformin (44). Metformin can also reduce the secretion of IL-6 by alveolar macrophages, thereby reducing pulmonary thrombosis in mice (45). Therefore, the use of metformin is beneficial in patients with T2DM who have COVID-19; we suggest the use of metformin for hypoglycemic treatment in those patients.

Sulfonylurea is a well-established oral hypoglycemic drug. Because of its precise hypoglycemic effect, good quality, and low price, it is both convenient and practical for many patients with diabetes. Five studies have reported the risk of mortality during sulfonylurea treatment in patients with COVID-19. Kim et al. (16) reported a 1:1 propensity cohort study, which revealed no difference in mortality (OR, 0.84; 95% CI, 0.23–3.09) between sulfonylurea users and non-users. Similarly, Wargny et al. (25) reported no difference in mortality (OR, 0.83; 95% CI, 0.67–1.03) between sulfonylurea users and non-users over a 28-day interval. Our findings are surprising because in-hospital use of sulfonylurea has been associated with lower mortality. The specific underlying mechanism is unclear. If islet function is acceptable, sulfonylurea drugs can be considered for hypoglycemic treatment in patients with T2DM who have COVID-19. However, sulfonylurea drugs can easily cause hypoglycemia; therefore, the use of sulfonylurea drugs in patients with severe COVID-19 requires careful blood glucose monitoring.

Our meta-analysis did not show any relationship between DPP-4 inhibitor use and mortality outcomes in patients with T2DM who had COVID-19. This is consistent with the previous comprehensive meta-analysis which showed that DPP-4 inhibitor did not alter the mortality from COVID-19 (46). Fadini et al. (27) reported an Italian case-control study involving 85 T2DM patients hospitalized with COVID-19; in that study, DPP-4 inhibitor treatment in nine patients was not associated with COVID-19 mortality. However, the small number of samples was an important limitation of that study deficiency. In the CORONADO study, a large-scale, nationwide observational study of 1317 T2DM patients with COVID-19, no differences in 7-day or 28-day mortality were observed between DDP-4 inhibitor users and non-users (24, 25). Similarly, in a study of 3818 patients with COVID-19, Strollo et al. (47) suggested that the pharmacological effects of DPP-4 inhibitors might not influence SARS-CoV-2 infection and COVID-19 progression. However, Mirani et al. (21) reported a decline in fatalities among DPP-4 inhibitor users, compared with non-users. A retrospective case-control study by Solerte et al. (26) showed that, compared with insulin therapy alone, patients who received sitagliptin plus insulin showed a >50% relative mortality reduction. However, Nauck et al. (48) reported some limitations regarding the studies by Mirani et al. and Solerte et al. In conclusion, DPP-4 inhibitors may slow the progression of acute respiratory complications in T2DM patients with COVID-19 (49). Although DPP-4 inhibitors are presumed to interact with the MERS-CoV receptor (49), which has characteristics similar to the SARS-CoV-2 receptor, these drugs have not been confirmed to interact with the SARS-CoV-2 receptor.

Viral infection can worsen hyperglycemia in patients with diabetes; it may induce acute diabetes complications. Hyperglycemia can cause immune abnormalities that aggravate infection, hypoxia, and overall COVID-19. Insulin is consistently the first choice for emergency treatment. Therefore, many experts recommend insulin for T2DM patients with SARS-CoV-2 infections (50, 51). Previous clinical studies regarding patients with diabetes who had sepsis, the 30-day mortality rate was higher among those receiving insulin treatment than among those receiving oral antidiabetic agents (52). Another study showed that insulin use led to greater mortality in 6104 intensive care unit patients (53). These findings suggest that glycemic control with insulin therapy leads to greater mortality risk in critically ill patients, possibly by promoting inflammation. Similarly, a study by Yu et al. (29) suggested that mortality was significantly greater in patients with T2DM who had COVID-19 and received insulin, compared with those who did not receive insulin (HR, 7.70; 95% CI, 4.22–14.05). The conclusion was still valid when analyzing the subgroups established by propensity score matching and different baseline characteristics or severity stratification. As shown in **Figure 5**, insulin use tended to be associated with greater mortality in patients with COVID-19. Although the underlying mechanism is unclear, our meta-analysis results suggest the need for careful assessment of the benefits and potential adverse effects of insulin therapy for patients with COVID-19.

To the best of our knowledge, this study is the first to compare mortality risks among various antidiabetic agents for the treatment of patients with T2DM who have COVID-19. We found that metformin and sulfonylurea treatments led to reduced mortality risk, while insulin was significantly associated with greater mortality. However, DPP-4 inhibitors showed no relationship with mortality risk in patients with T2DM who had COVID-19.

However, this study had some limitations. First, since all studies are observational, this meta-analysis is only hypothesis-generating. Prospective randomized studies are required to draw conclusions and to give treatment advice for patients with T2DM who have COVID-19. Second, although the included study findings were adjusted to control for major potential confounding factors, the various degrees of residual confounding bias, including the different severity of diabetes itself, could not be fully excluded. Additionally, comorbidity such as dementia or Parkinson’s disease may be a confounder for elderly patients related to mortality (54, 55). Finally, bias may have been present because of variable research quality among the included studies.

## Conclusion

Metformin and sulfonylurea treatments could be associated with reduced mortality risk, while insulin treatment could be associated with enhanced mortality risk, in patients with T2DM who had COVID-19. However, DPP-4 inhibitor treatment could not be associated with mortality risk in these patients. The results of this meta-analysis should be interpreted carefully because of the limitations of included studies, although the effects of sulfonylurea and DPP-4 inhibitors should be more fully evaluated in subsequent studies. Further larger trials should also be done to confirm these results and especially other diabetes drugs including SGLT2 inhibitors and DPP-4 inhibitors.

## Data Availability Statement

The original contributions presented in the study are included in the article/supplementary material. Further inquiries can be directed to the corresponding authors.

## Author Contributions

CK, YZ, and FH: Conceptualization, methodology, and writing – original draft preparation. TY and QX: Data curation and investigation. XS and NH: Supervision, writing – reviewing and editing, and funding acquisition. All authors contributed to the article and approved the submitted version.

## Funding

This work was supported by the National Natural Science Foundation of China (81870593), Natural Science Foundation of Shandong Province of China (ZR2020MH106), Shandong Province Higher Educational Science and Technology Program for Youth Innovation (2020KJL004) and Quality Improvement of Postgraduate Education in Shandong Province (SDYAL19156).

## Conflict of Interest

The authors declare that the research was conducted in the absence of any commercial or financial relationships that could be construed as a potential conflict of interest.

## Publisher’s Note

All claims expressed in this article are solely those of the authors and do not necessarily represent those of their affiliated organizations, or those of the publisher, the editors and the reviewers. Any product that may be evaluated in this article, or claim that may be made by its manufacturer, is not guaranteed or endorsed by the publisher.
